# The Deubiquitinating Enzyme UCHL1 Promotes Radiotherapy Resistance in Glioma by Stabilizing Keratin 18

**DOI:** 10.3390/ijms27219875

**Published:** 2026-11-04

**Authors:** Hanlu Wang, Rujuan Zheng, Qian Liu, Xinyuan Ding, Ying Zhao, Jianyu Xu, Ruze Tang, Lulu Chen, Xi Chen, Hui Shi, Huiling Zhang, Wenjuan Wang

**Affiliations:** 1 Department of Pharmacy, Children’s Hospital of Soochow University, Suzhou 215025, China; 15737845171@163.com (H.W.); zhengrujuan0416@163.com (R.Z.); zy3434@suda.edu.cn (Y.Z.); 19558965362@163.com (X.C.); shihui11062000@163.com (H.S.); 2 College of Pharmaceutical Sciences, Soochow University, Suzhou 215123, China; zhanghuiling@suda.edu.cn; 3 Department of Pathology, The Affiliated Suzhou Hospital of Nanjing Medical University, Suzhou Municipal Hospital, Gusu School of Nanjing Medical University, Suzhou 215001, China; qianliu1990@163.com; 4 Department of Pharmacy, Medical Science and Technology Innovation Center, The Affiliated Suzhou Hospital of Nanjing Medical University, Suzhou Municipal Hospital, Gusu School of Nanjing Medical University, Suzhou 215001, China; aladdine@163.com (X.D.); xiaxicangmu@163.com (J.X.); 5 Department of Surgical Oncology, Children’s Hospital of Soochow University, Suzhou 215025, China; tang1826010@163.com (R.T.); lulu_chen@foxmail.com (L.C.)

**Keywords:** glioma, radioresistance, stemness, ubiquitin carboxy-terminal hydrolase L1, keratin 18

## Abstract

Maintaining stemness in glioma stem cells (GSCs), coupled with robust DNA damage repair capacity, is a key determinant of radioresistance in glioma. Although the deubiquitinating enzyme ubiquitin carboxy-terminal hydrolase L1 (UCHL1) has been implicated in regulating tumor cell functions and facilitating malignant progression, its specific role in sustaining GSC stemness and in promoting radioresistance remains poorly understood. In this study, we combined secondary analyses of public datasets with in–house experimental investigations. Specifically, we first analyzed *UCHL1* expression and its prognostic significance in glioma using the GEO2R, GEPIA2, and CGGA databases. Radiosensitivity, DNA repair capability, and GSC stemness were evaluated using clonogenic survival, comet, sphere formation, extreme limiting dilution assay, and Western blot analyses. To identify UCHL1–interacting proteins, co-immunoprecipitation coupled with mass spectrometry was performed, followed by deubiquitination and rescue experiments to validate its substrates and functional dependence. Our results revealed that UCHL1 was markedly overexpressed in radioresistant glioblastoma cells and glioma tissues, and its expression levels positively correlated with poor prognosis in patients with glioma. *UCHL1* knockdown attenuated the sphere-forming capacity and self–renewal frequency of GSCs and impaired the cellular response to DNA damage following irradiation, thereby attenuating the radioresistant phenotype. Mechanistically, UCHL1 stabilizes keratin 18 (KRT18) protein via deubiquitination. The effects of UCHL1 on radioresistance and stemness properties were largely diminished upon *KRT18* knockdown, accompanied by reduced expression of the stemness marker SOX2. Furthermore, *KRT18* knockdown attenuated the radioresistant phenotype induced by *UCHL1* overexpression. Collectively, these findings suggest a mechanistic axis through which UCHL1 may regulate GSC stemness and radioresistance and identify UCHL1 as a candidate therapeutic target for reversing therapeutic resistance in glioma, warranting further validation.

## 1. Introduction

Glioma represents the most prevalent and aggressive primary malignant tumor of the central nervous system [1,2]. Although multimodal treatment encompassing surgical resection followed by chemoradiotherapy has been established as the standard of care regimen, therapeutic resistance to ionizing radiation remains a pivotal determinant of the dismal prognosis and frequent tumor recurrence observed in glioma patients [3,4,5]. Conventional chemotherapeutic agents also play an important role in the treatment of central nervous system tumors; however, they are frequently associated with severe adverse effects such as neurotoxicity [6], underscoring the urgent need for more precise therapeutic strategies with lower toxicity. Hence, a comprehensive understanding of the molecular underpinnings of radioresistance is imperative for the design of effective radiosensitization strategies.

Radiotherapy predominantly exerts its cytotoxic effects through the induction of lethal DNA lesions [7]. In response, tumor cells frequently exploit their robust DNA damage repair (DDR) capacity, which constitutes the principal mechanism driving resistance to radiation therapy [8]. Within the heterogeneous glioma tumor mass, a subpopulation of glioma stem cells (GSCs) possesses superior DDR proficiency and self-renewal capabilities, making them the primary drivers of tumor persistence and major contributors to profound radioresistance [9,10]. GSCs evade radiation-induced cell death by activating DNA damage checkpoint kinases, including ATM and ATR [11], and by upregulating repair pathways, such as homologous recombination (HR) and non-homologous end joining (NHEJ) [12,13]. Furthermore, the immunosuppressive microenvironment and metabolic reprogramming characteristic of GSC niches further potentiate their resistance to radiotherapy [14]. In recent years, accumulating evidence has underscored the substantial roles of epigenetic regulation and post-translational protein modifications in sustaining GSC stemness and modulating DDR, thereby offering novel conceptual frameworks for dissecting radioresistance mechanisms [15,16]. Nevertheless, the precise molecular events remain largely elusive.

The ubiquitin–proteasome system (UPS) constitutes a major intracellular proteolytic pathway that governs a wide spectrum of fundamental cellular processes—including cell cycle progression, apoptosis, DDR, and stem cell differentiation—through precise modulation of critical protein stability [17]. Deubiquitinases (DUBs) counteract proteasomal degradation by removing ubiquitin conjugates from substrate proteins [18], thereby profoundly influencing the stabilization of key oncoproteins, the regulation of DDR cascades, and the maintenance of cancer stem cell properties [19]. Prior investigations have revealed that USP28 sustains GSC stemness and drives gliomagenesis via c-Myc stabilization [20]; similarly, RNF8 and RNF168 facilitate the accumulation of DNA repair factors such as BRCA1 at DNA double-strand break sites, thereby enhancing DDR capacity and conferring radioresistance in tumor cells [21]. Although the UPS is increasingly recognized as a regulator of DNA damage responses and radioresistance in other cancers [22], the specific contribution of DUBs remains less defined. As a member of the ubiquitin C-terminal hydrolase (UCH) family, ubiquitin C-terminal hydrolase L1 (UCHL1) primarily functions as a deubiquitinating enzyme that stabilizes diverse oncoproteins [23]. UCHL1 is highly expressed in multiple malignancies, including prostate cancer, cervical squamous cell carcinoma, and triple-negative breast cancer, where it promotes malignant progression by modulating cancer stem cell traits, proliferation, migration, and invasion [24,25,26]. Our previous work has also established the involvement of UCHL1 in tumor drug resistance and metastatic dissemination [27,28]. However, the specific contribution of UCHL1 to radioresistance in glioma remains to be fully characterized.

In this study, we identify UCHL1 as a mediator of therapy resistance that may help regulate stemness maintenance and the cellular response to DNA damage. We further characterize its functional and mechanistic roles and identify keratin 18 (KRT18) as a previously unrecognized deubiquitination substrate of UCHL1. Mechanistically, UCHL1 removes K48–linked polyubiquitin chains from KRT18, attenuating its proteasomal degradation and preserving KRT18 stability. This UCHL1–KRT18 axis, in turn, may help sustain stemness properties and promote radioresistance by upregulating SOX2 expression. Collectively, our results suggest UCHL1 as a candidate therapeutic target and delineate a mechanistic framework for further investigations to sensitize glioma to radiation therapy.

## 2. Results

### 2.1. UCHL1 Expression Positively Correlates with Radioresistance in Glioma

**Figure 1:**
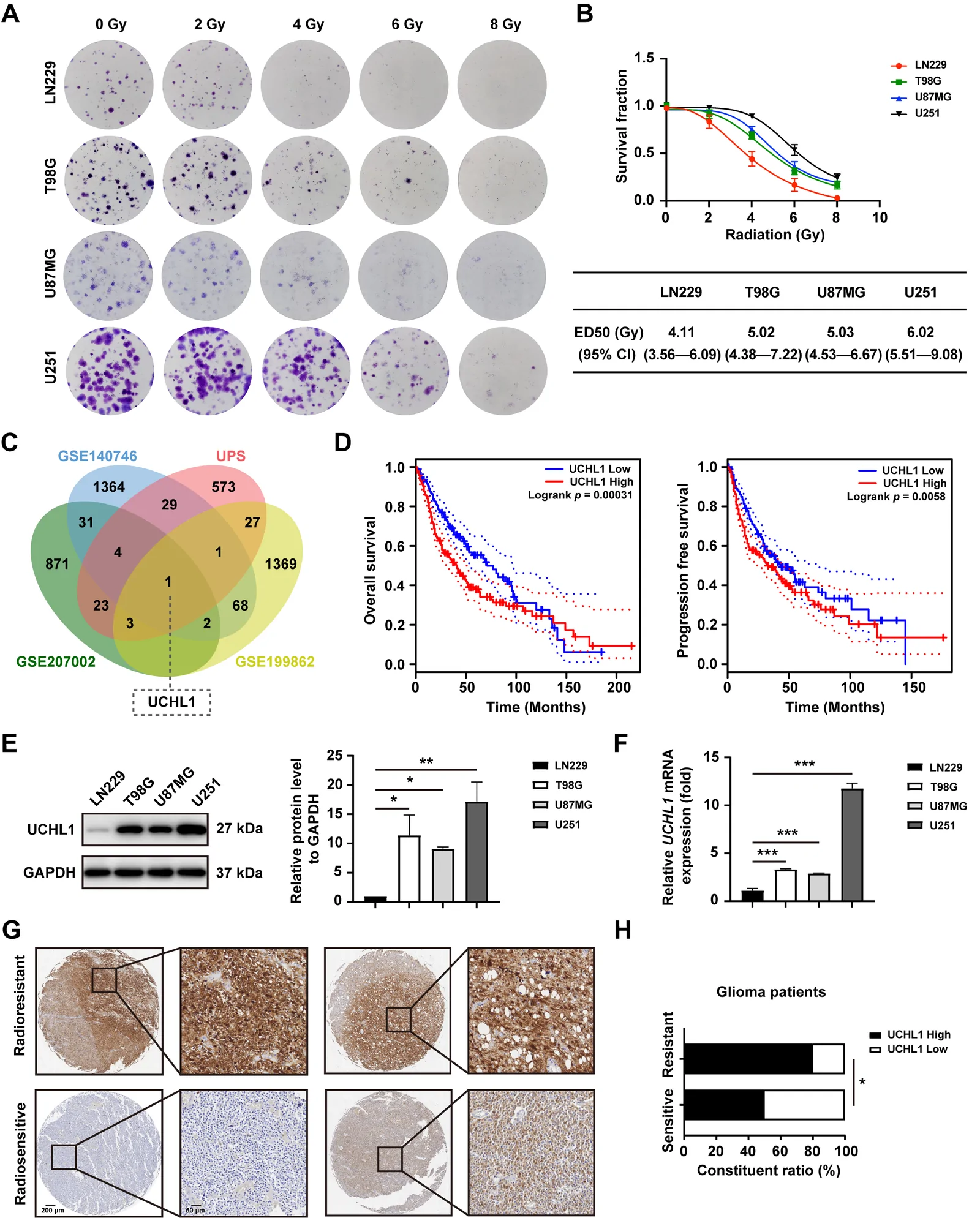
**UCHL1 expression positively correlates with radioresistance in glioma.** (**A**) Clonogenic survival assays evaluating the colony–forming capacity of the four indicated GBM cell lines following exposure to single–dose irradiation. (**B**) Quantification of colony numbers and median effective dose (ED50, Gy) following irradiation. ED50 values are shown with 95% confidence intervals (CI), derived from three independent experiments. (**C**) Venn diagram depicting the overlap between differentially expressed genes derived from radioresistance-related GEO datasets (GSE199862, GSE207002, and GSE140746) and ubiquitin–proteasome system (UPS)–related genes. (**D**) Kaplan–Meier survival curves illustrating the correlation between *UCHL1* expression and overall survival (*p* = 0.00031) as well as progression–free survival (*p* = 0.0058) in glioma patients. (**E**) Western blot analysis of UCHL1 protein expression in LN229, T98G, U87MG, and U251 cells (*n* = 3). (**F**) qRT–PCR analysis of *UCHL1* mRNA levels in the indicated cell lines, normalized to GAPDH (*n* = 3). (**G**) Representative immunohistochemical (IHC) staining of UCHL1 in radiosensitive and radioresistant glioma tissue specimens. (**H**) Quantitative comparison of UCHL1 expression levels between radioresistant and radiosensitive glioma tissue specimens. * *p* < 0.05, ** *p* < 0.01, *** *p* < 0.001.

To assess the differential radiosensitivity of glioblastoma (GBM) cell lines, clonogenic survival assays were performed following exposure to graded doses of irradiation (0–8 Gy) in LN229, T98G, U87MG and U251 cells. As shown in Figure 1A,B, U251 cells exhibited a higher median effective dose (ED50: 6.02 Gy, 95% CI: 5.51–9.08 Gy), whereas LN229 cells displayed the lowest ED50 (4.11 Gy, 95% CI: 3.56–6.09 Gy), indicating that U251 cells are relatively radioresistant, in contrast to the radiosensitive phenotype of LN229 cells. Given the established role of protein homeostasis in tumor radioresistance, we examined the contribution of two major proteolytic pathways to the radiosensitivity of GBM cells. Radioresistant U251 cells were treated with MG132 or chloroquine (CQ) combined with irradiation, and clonogenic survival was subsequently evaluated. MG132 treatment resulted in a dose–dependent increase in colony formation efficiency under irradiated conditions, whereas CQ treatment exerted no significant effect on clonogenic survival regardless of irradiation (Figure S1A,B). These observations indicate that UPS–mediated protein homeostasis critically contributes to maintaining the radioresistant phenotype of GBM cells. Based on these findings, we conducted a bioinformatics screen to identify UPS–related genes associated with radioresistance. By intersecting differentially expressed genes derived from glioma radioresistance-related datasets in the GEO database (GSE199862, GSE207002, and GSE140746) with the UPS–related genes from UniProt, we identified the deubiquitinating enzyme UCHL1 as the UPS member consistently upregulated across the radioresistant groups (Figure 1C), suggesting a potential role for UCHL1 in modulating radioresistance through the UPS pathway.

To further assess the clinical relevance of UCHL1, Kaplan–Meier survival analysis using the GEPIA2 database showed that glioma patients with high *UCHL1* expression exhibited significantly shorter overall survival and progression–free survival than those with low *UCHL1* expression (Figure 1D). Moreover, UCHL1 expression was significantly elevated in recurrent tumors relative to primary tumors (Figure S1C, *p* = 0.029). Having identified UCHL1 as a candidate radioresistance–associated gene, we next asked whether its expression tracks with radiosensitivity across glioma cell lines. To this end, we first analyzed UCHL1 expression across 15 commonly used glioma cell lines in the Cancer Cell Line Encyclopedia (CCLE) database (Figure S1D), and on this basis selected U251 and U87MG (relatively high *UCHL1* expression) as well as T98G and LN229 (relatively low *UCHL1* expression) for validation. Subsequently, protein and mRNA levels of UCHL1 were measured in four GBM cell lines by Western blot and qRT–PCR. The results showed that UCHL1 protein and mRNA levels were lowest in the radiosensitive LN229 cells and highest in the radioresistant U251 cells (Figure 1E,F); therefore, U251 was selected for *UCHL1* knockdown and LN229 for *UCHL1* overexpression in subsequent experiments. Finally, UCHL1 expression in clinical glioma specimens was validated by IHC. Semi–quantitative scoring showed that UCHL1 levels were higher in the radioresistant group than in the radiosensitive group (Figure 1G,H, Figure S1E). Collectively, these results indicate that UCHL1 is highly expressed in radioresistant GBM cells and glioma patient tissues, suggesting it may help regulate radioresistance in glioma.

### 2.2. UCHL1 Regulates Radiosensitivity and DNA Repair Capacity in GBM Cells

**Figure 2:**
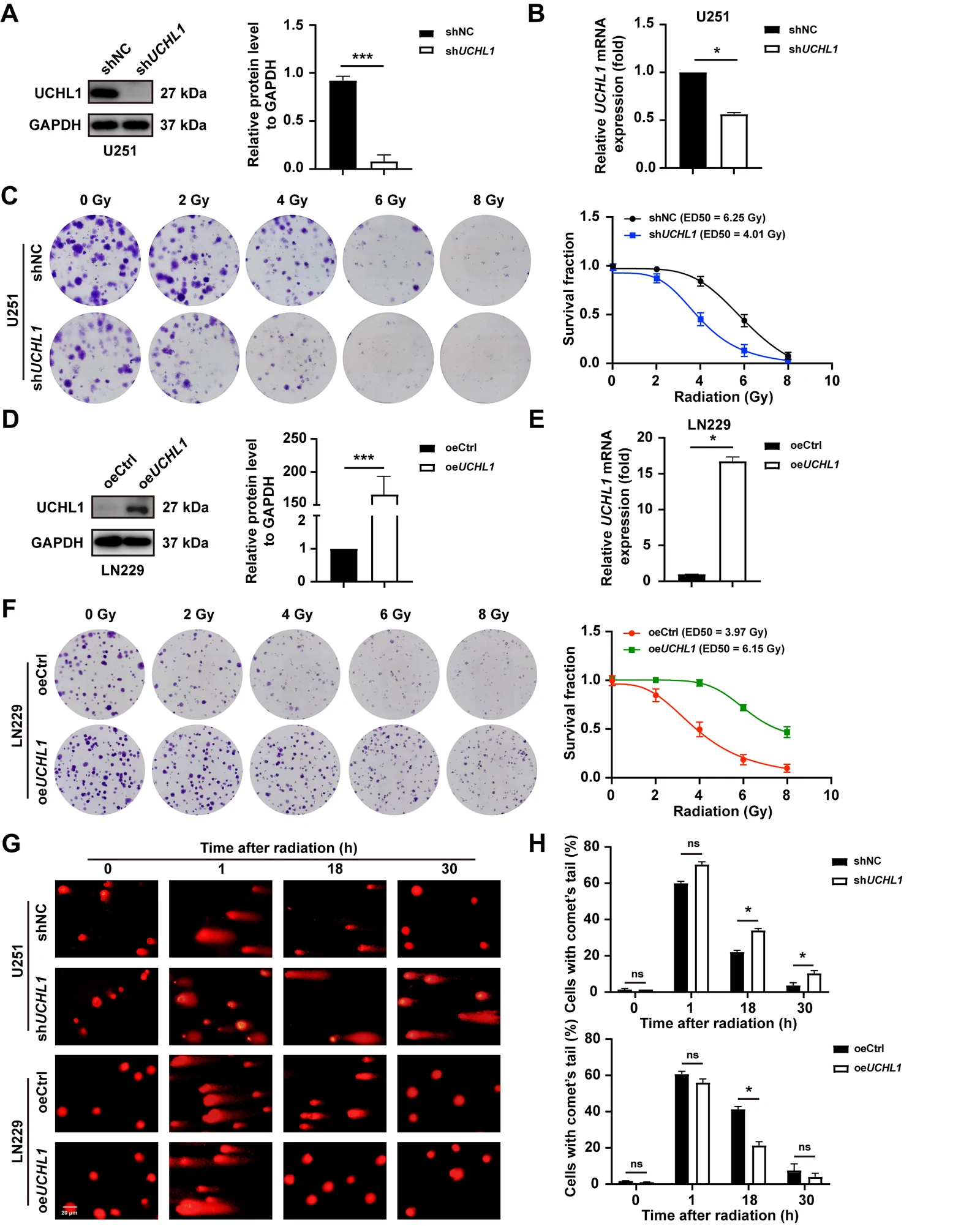
**UCHL1 regulates radiosensitivity and DNA repair capacity in GBM Cells.** (**A**) Western blot validation of UCHL1 protein knockdown efficiency in U251 cells transduced with sh*UCHL1* or sh*NC* lentivirus (*n* = 3). (**B**) qRT–PCR quantification of *UCHL1* mRNA levels following knockdown in U251 cells, normalized to GAPDH (*n* = 3). (**C**) Clonogenic survival assays evaluating the colony–forming capacity of U251 sh*NC* and U251 sh*UCHL1* cells following graded doses of irradiation (*n* = 3). (**D**) Western blot confirmation of UCHL1 protein overexpression in LN229 cells transduced with Flag–*UCHL1* or empty vector lentivirus (*n* = 3). (**E**) qRT-PCR analysis of *UCHL1* mRNA levels following ectopic overexpression in LN229 cells, normalized to GAPDH (*n* = 3). (**F**) Clonogenic survival assays evaluating the colony-forming capacity of LN229 oe*Ctrl* and LN229 oe*UCHL1* cells after irradiation (*n* = 3). (**G**,**H**) Comet assay evaluating the cellular response to DNA damage in U251 (**G**) and LN229 (H) cells following irradiation. Single–cell gel electrophoresis was performed at 1, 18, and 30 h post-irradiation (6 Gy) to monitor residual DNA damage. Data were derived from three independent experiments. * *p* < 0.05, *** *p* < 0.001, ns = not significant.

To investigate the functional role and underlying mechanism of UCHL1 in glioma radioresistance, we first established U251 cells with stable *UCHL1* knockdown (U251–sh*UCHL1*) using lentivirus-mediated shRNA interference, with negative–control–transfected cells (U251-sh*NC*) serving as controls. Knockdown efficiency was confirmed by Western blot and qRT–PCR (Figure 2A,B). Clonogenic survival assays following graded irradiation revealed that the surviving fraction of U251–sh*UCHL1* cells was reduced in a dose–dependent manner, accompanied by markedly decreased ED50 values (from 6.25 Gy, 95% CI: 5.64–10.63 Gy to 4.01 Gy, 95% CI: 3.47–4.78 Gy), indicating that *UCHL1* silencing effectively attenuates radioresistance of GBM cells (Figure 2C). Conversely, *UCHL1* overexpression in LN229 cells enhanced their radioresistance (Figure 2D–F, ED50 values from 3.97 Gy, 95% CI: 3.36–4.44 Gy to 6.15 Gy, 95% CI: 5.71–10.82 Gy). Comet assays were performed to further evaluate the cellular response to DNA damage. At 1 h post–irradiation, the extent of initial DNA damage was comparable between the control and knockdown groups. However, at 18 h and 30 h post–irradiation, the proportion of comet-positive cells in the knockdown group was significantly higher than that in the control group, indicating that *UCHL1* deficiency is associated with altered DNA damage response kinetics (Figure 2G). In contrast, LN229 cells overexpressing *UCHL1* showed an attenuated DNA damage response, as reflected by a lower proportion of comet–positive cells (Figure 2H).

To further substantiate the association between UCHL1 and radioresistance, radioresistant sublines U251R and LN229R were established through stepwise dose–escalation irradiation. Morphological examination revealed that these resistant cells acquired invasive characteristics, including spindle-shaped morphological changes and increased pseudopodia formation (Figure S2A,B). Clonogenic assays demonstrated that the survival fractions of U251R and LN229R cells were higher than those of their parental counterparts, with substantially elevated ED50 values (Figure S2C,D). Comet assays showed that the DNA repair capacity was markedly enhanced in radioresistant cells relative to parental cells at the 18 h repair time point (Figure S2E,F). Western blot further confirmed that UCHL1 protein levels were significantly upregulated in both U251R and LN229R cells compared with their parental counterparts (Figure S2G,H). Collectively, these findings indicate that UCHL1 modulates the cellular response to DNA damage, contributing to GBM cells’ radioresistance and supporting UCHL1 as a potential therapeutic target to counteract radioresistance in glioma.

### 2.3. UCHL1 Maintains Stemness Properties and Self–Renewal Capacity to Promote Radioresistance in GBM Cells

**Figure 3:**
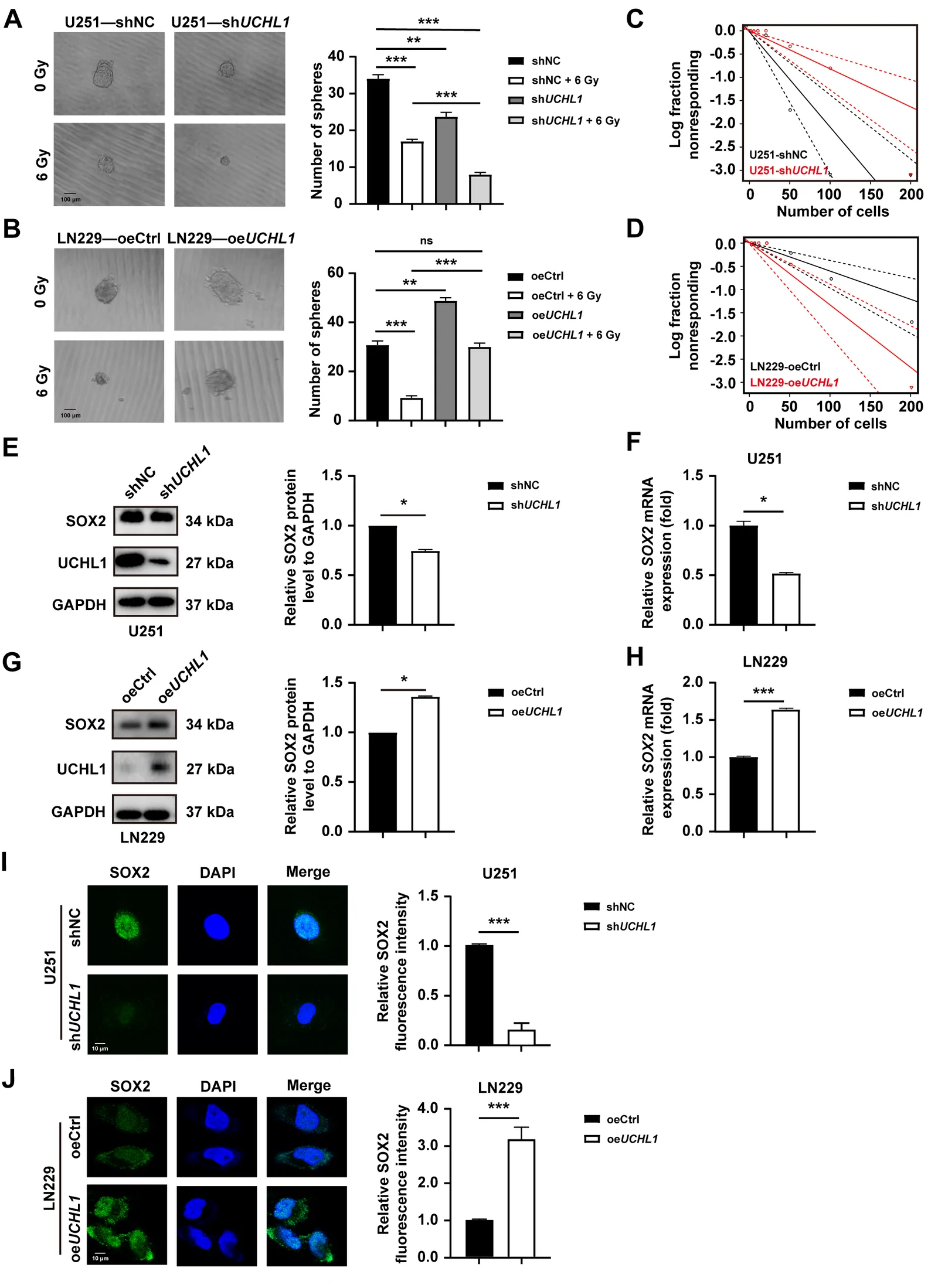
**UCHL1 maintains stemness properties and self-renewal capacity to promote radioresistance in GBM cells.** (**A**,**B**) Sphere formation assays examining sphere diameter and formation number in U251 (**A**) and LN229 (**B**) cells under basal conditions and following 6 Gy irradiation. Scale bar, 100 μm (*n* = 10). (**C**,**D**) ELDA quantifying the self-renewal frequency of U251–shNC and U251–sh*UCHL1* cells (**C**) and LN229–oeCtrl and LN229–oe*UCHL1* cells (**D**). (**E**,**G**) Western blot analysis of SOX2 protein expression in the indicated U251 (**E**) and LN229 (**G**) cells (*n* = 3). (**F**,**H**) qRT–PCR analysis of *SOX2* mRNA levels in the indicated U251 (**F**) and LN229 (**H**) cells, normalized to GAPDH (*n* = 3). (**I**,**J**) Immunofluorescence staining of SOX2 in U251 (**I**) and LN229 (**J**) cells. Scale bar, 10 μm (*n* = 3). * *p* < 0.05, ** *p* < 0.01, *** *p* < 0.001, ns = not significant.

As cancer stem cells are known to possess robust DNA damage repair capacity [9], we investigated whether UCHL1 modulates stemness maintenance following radiotherapy using sphere formation assays. *UCHL1* knockdown in U251 cells significantly reduced both basal sphere diameter and the number of sphere formations relative to control cells, and these parameters were further diminished after exposure to irradiation, suggesting that *UCHL1* deficiency compromises stemness maintenance in stem–cell–like GBM cells (Figure 3A). Conversely, *UCHL1* overexpression in LN229 cells significantly enhanced sphere–forming capacity under both basal and post–irradiation conditions (Figure 3B). To quantitatively assess self–renewal frequency, extreme limiting dilution analysis (ELDA) was performed, revealing decreased sphere formation frequency from 1/128 to 1/512 in the U251–sh*UCHL1* cells, whereas overexpressed LN229 cells increased the frequency from 1/256 to 1/64 (Figure 3C,D). These findings indicate that UCHL1 regulates radioresistance by maintaining stemness properties. We further evaluated stemness characteristics in established radioresistant GBM sublines (U251R and LN229R). Sphere formation assays revealed that, under basal conditions, the radioresistant cells exhibited significantly higher sphere-forming capacity compared with their parental counterparts, and retained this enhanced capacity following irradiation, whereas parental cells showed a pronounced reduction (Figure S3A,B).

Given the observed changes in stemness maintenance and self–renewal capacity upon UCHL1 modulation, we next examined the expression of the stemness–associated marker SOX2. Western blot and qRT–PCR analyses revealed a positive correlation between UCHL1 and SOX2 expression: *UCHL1* knockdown significantly reduced both SOX2 protein and mRNA levels, whereas *UCHL1* overexpression exerted opposite effects (Figure 3E–H). Immunofluorescence staining further confirmed this expression pattern (Figure 3I,J). We assessed SOX2 mRNA abundance in radioresistant cells compared with parental cells to determine whether this regulatory relationship exists. The results further confirmed that SOX2 transcript levels were significantly elevated in radioresistant cells (Figure S3C,D). Collectively, these results suggest that UCHL1 may contribute to radioresistance in GBM cells by positively regulating SOX2–mediated stemness maintenance and self–renewal capacity.

### 2.4. KRT18 Is Identified as a Novel Interacting Protein of UCHL1

**Figure 4:**
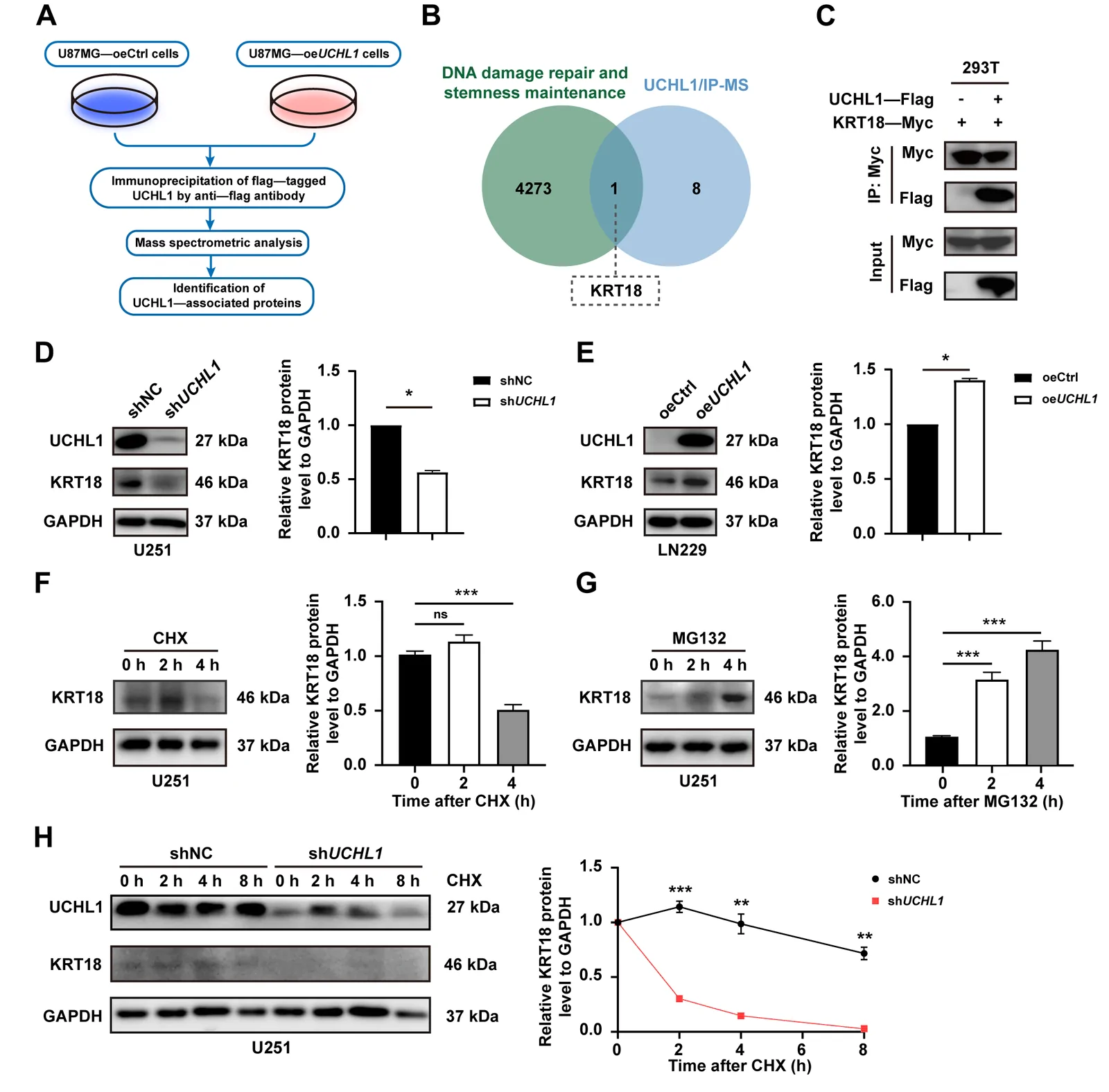
**KRT18 is identified as a novel interacting protein of UCHL1.** (**A**) Workflow of immunoprecipitation mass spectrometry (IP–MS) used to screen for UCHL1 interacting proteins. (**B**) Venn diagram illustrating the intersection of UCHL1–interacting proteins identified by IP–MS with genes associated with DNA damage repair and stemness maintenance from the Genecards database. (**C**) Co–immunoprecipitation (Co–IP) analysis validating the interaction between KRT18 and UCHL1. (**D**) Western blot analysis of KRT18 protein levels in U251–shNC and U251–sh*UCHL1* cells (*n* = 3). (**E**) Western blot analysis of KRT18 protein levels in LN229–oeCtrl and LN229–oe*UCHL1* cells (*n* = 3). (**F**) U251 cells were treated with cycloheximide (CHX, 100 ng/mL) for the indicated times (0, 2, and 4 h), and KRT18 protein levels were analyzed by Western blotting (*n* = 3). (**G**) U251 cells were treated with MG132 (10 μM) for the indicated times (0, 2, and 4 h), and KRT18 protein levels were examined by Western blotting (*n* = 3). (**H**) U251 cells transfected with shNC or sh*UCHL1* were treated with CHX (100 ng/mL) for the indicated times (0, 2, 4, and 8 h), followed by Western blot analysis of KRT18 protein levels (*n* = 3). * *p* < 0.05, ** *p* < 0.01, *** *p* < 0.001, ns = not significant.

To dissect the molecular interaction network of UCHL1 in glioma, we established a *UCHL1*–Flag overexpression system in U87MG cells and immunoprecipitated UCHL1–associated protein complexes for mass spectrometry analysis (Figure 4A). Nine differentially associated proteins were identified, and their intersection with 4274 genes annotated for “DNA damage repair” and “stemness maintenance” in the Genecards database yielded KRT18 as the UCHL1–interacting protein simultaneously linked to both biological processes (Figure 4B). The interaction between UCHL1 and KRT18 was validated using Co–IP assays in 293T cells. Anti–Flag antibody specifically co–precipitated exogenous *KRT18*–Myc, whereas no appreciable binding was observed in control groups, confirming a direct interaction between UCHL1 and KRT18 (Figure 4C).

Western blot analysis revealed that KRT18 expression was decreased in U251–sh*UCHL1* cells and increased in LN229–oe*UCHL1* cells to examine the regulatory effect of UCHL1 on KRT18 protein levels (Figure 4D,E). To elucidate the underlying mechanism, cycloheximide (CHX) chase assays were performed to monitor KRT18 protein decay kinetics, which showed a time–dependent reduction in KRT18 protein levels (Figure 4F). MG132 intervention experiments further confirmed that KRT18 is degraded via the ubiquitin–proteasome pathway (Figure 4G). In U251–sh*UCHL1* cells, combined CHX treatment significantly shortened the half–life of KRT18 protein (Figure 4H), indicating that UCHL1 sustains KRT18 protein stability through its deubiquitinating activity. These results identify KRT18 as a novel UCHL1–interacting protein and demonstrate that UCHL1 maintains KRT18 stability by inhibiting its ubiquitin–proteasome pathway-mediated degradation.

### 2.5. UCHL1 Stabilizes KRT18 Through K48-Linked Deubiquitination

**Figure 5:**
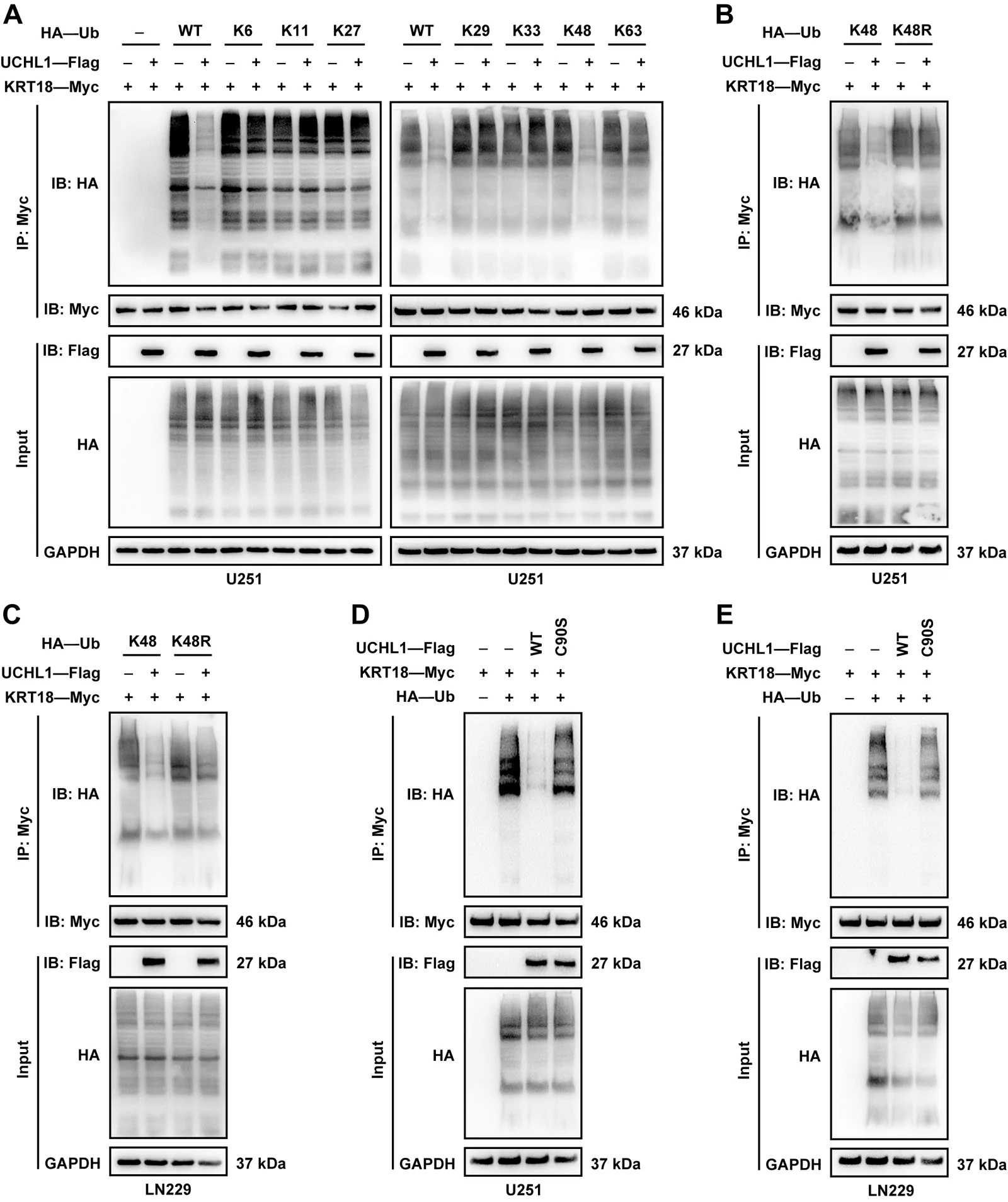
**UCHL1 stabilizes KRT18 through K48-linked deubiquitination.** (**A**) Assessment of the ubiquitin linkage type involved in UCHL1–mediated regulation of KRT18. U251 cells co–transfected with the indicated plasmids were treated with MG132 (20 μM) for 8 h, and ubiquitinated KRT18 levels were detected by a ubiquitination assay. (**B**,**C**) Analysis of ubiquitin linkage specificity of KRT18 mediated by UCHL1. U251 (**B**) and LN229 (**C**) cells were co–transfected with the indicated lysine–to–arginine (KR) ubiquitin mutants, and ubiquitinated KRT18 levels were examined by ubiquitination assay. K, lysine; R, arginine. (**D**,**E**) Co-immunoprecipitation (Co-IP) analysis of UCHL1–mediated deubiquitination of KRT18. U251 (**D**) and LN229 (**E**) cells were co-transfected with wild–type UCHL1 (UCHL1–WT) or the catalytically inactive mutant UCHL1–C90S, followed by assessment of KRT18 ubiquitination levels. The C90S mutation inactivates the deubiquitinating enzyme activity of UCHL1.

To determine whether UCHL1 regulates KRT18 stability through deubiquitination, we first assessed the effect of UCHL1 on the specific ubiquitin linkage type of KRT18. *KRT18* co–transfection with various HA-tagged ubiquitin mutants, followed by Co-IP, revealed that UCHL1 preferentially removed K48–linked ubiquitin chains from KRT18 (Figure 5A). We co–transfected KRT18 with HA–ubiquitin mutants in which individual lysine residues were substituted with arginine (KR mutants) to further delineate the specific lysine residues involved. The ubiquitination signal of the K48R mutant was resistant to UCHL1-mediated removal (Figure 5B,C), confirming that UCHL1 primarily cleaves K48-linked polyubiquitin chains on KRT18.

To verify whether UCHL1 regulates KRT18 ubiquitination in cells, we co–transfected *KRT18*–Myc, HA–Ub, and either wild–type (WT) or catalytically inactive (C90S) *UCHL1*–Flag into U251 and LN229 cells. Co–IP analyses showed that overexpressed wild–type UCHL1 markedly reduced the ubiquitination signal of KRT18, whereas the C90S mutant exhibited no detectable effect (Figure 5D,E), indicating that the deubiquitinating enzyme activity of UCHL1 is specifically required for KRT18 ubiquitination. Taken together, these findings indicate that UCHL1 specifically removes K48–linked polyubiquitin chains from KRT18 through its deubiquitinating enzyme activity, thereby inhibiting its proteasomal degradation and sustaining KRT18 protein stability.

### 2.6. KRT18 Is Required for UCHL1-Related Radioresistance in Glioma

**Figure 6:**
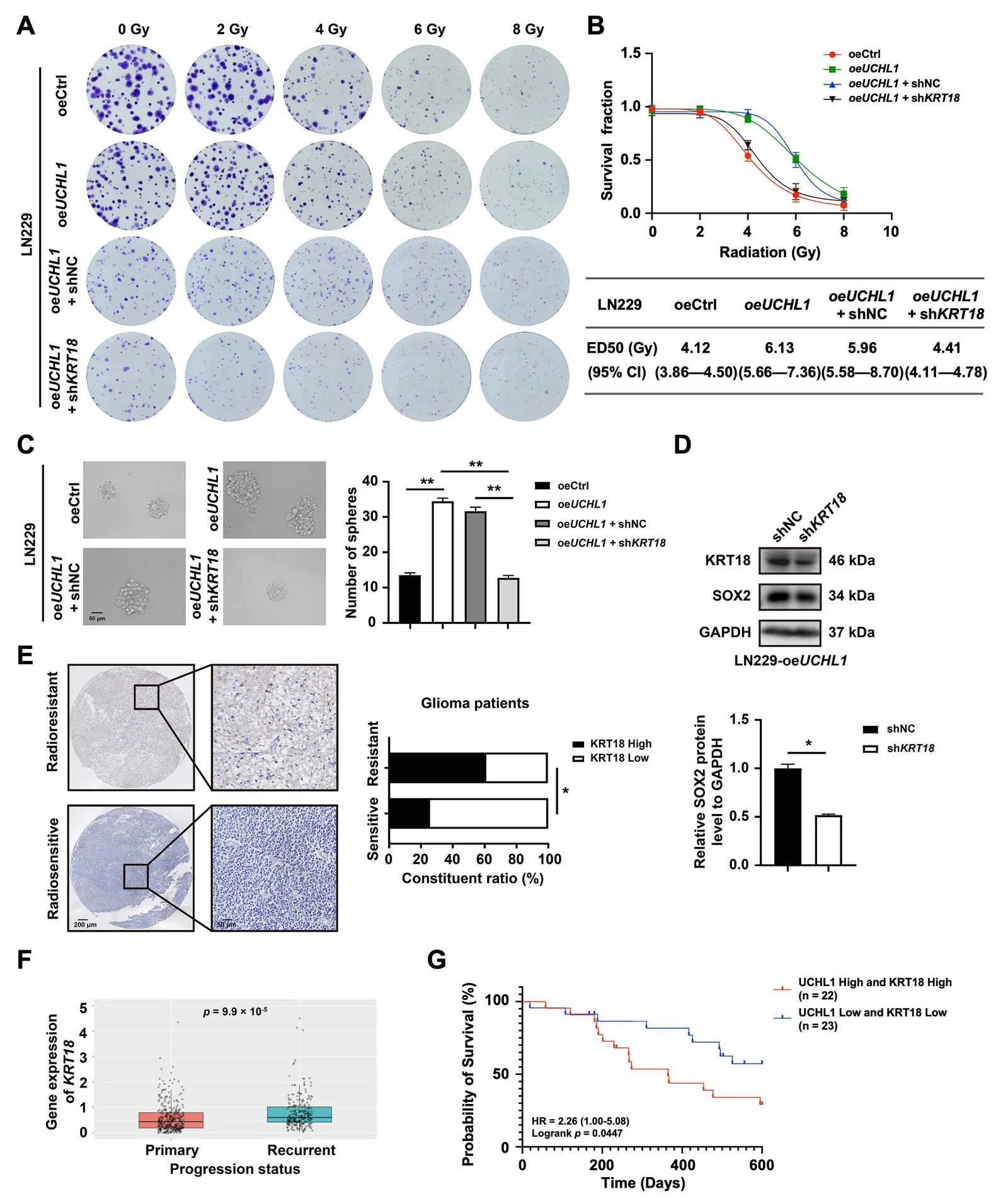
**KRT18 is required for UCHL1-related radioresistance in glioma.** (**A**,**B**) Clonogenic survival assays evaluating the radiosensitivity of LN229 oe*UCHL1* cells with sh*KRT18* or corresponding controls, with quantification of colony numbers and ED50. ED50 values are shown with 95% CI, derived from three independent experiments. (**C**) Sphere formation assay assessing the size and number of stem cell spheres upon *KRT18* knockdown in *UCHL1*–overexpressing GBM cells. Scale bar, 100 μm (*n* = 10). (**D**) Western blot analysis of KRT18 and SOX2 protein levels in LN229 oe*UCHL* cells (*n* = 3). (**E**) Representative IHC images of KRT18 expression in radiosensitive and radioresistant glioma tissue specimens, along with quantitative comparison of KRT18 expression levels between the two groups. (**F**) *KRT18* expression in primary versus recurrent glioma specimens from the CGGA database. (**G**) Kaplan–Meier survival analysis of the correlation between *UCHL1*–*KRT18* expression and overall survival in glioma patients, evaluated using the log-rank test. * *p* < 0.05, ** *p* < 0.01.

To examine the functional contribution of KRT18 to glioma radioresistance, we knocked down *KRT18* in LN229 cells overexpressing *UCHL1*. Clonogenic survival assays showed that *UCHL1* overexpression enhanced the clonogenic capacity of these cells, with the ED50 value increasing from 4.12 Gy (95% CI: 3.86–4.50 Gy) in the control group to 6.13 Gy (95% CI: 5.66–7.36 Gy). KRT18 knockdown attenuated this radioresistant phenotype, reducing the ED50 value to 4.41 Gy (95% CI: 4.11–4.78 Gy) (Figure 6A,B). Sphere formation assays further showed that *KRT18* knockdown diminished the enhanced sphere-forming capacity associated with *UCHL1* overexpression (Figure 6C). Western blotting revealed that *KRT18* knockdown also reduced SOX2 protein levels (Figure 6D), consistent with a possible involvement of KRT18 in SOX2–related stemness maintenance in the context of glioma radioresistance.

We next examined KRT18 expression in clinical glioma specimens using IHC. Semiquantitative IHC scoring showed that KRT18 expression was higher in the radioresistant group than in the radiosensitive group (Figure 6E). To assess the clinical relevance of KRT18 in glioma, we analyzed its expression in primary versus recurrent tumor samples from the CGGA database. *KRT18* expression was elevated in recurrent tumors compared with primary tumors (Figure 6F). Kaplan–Meier analysis using the GEPIA2 database showed that patients with high *KRT18* expression had shorter overall survival and progression–free survival than those with low *KRT18* expression (Figure S4A,B), suggesting an association between high *KRT18* expression and poor prognosis in glioma patients. Kaplan–Meier analysis of the correlation between combined *UCHL1/KRT18* expression and overall survival further showed that patients with high *UCHL1* and *KRT18* expression had shorter survival than those with low *UCHL1* and *KRT18* expression (Figure 6G, *p* = 0.0447). Collectively, these results indicate that KRT18 is overexpressed in radioresistant glioma patient tissues and suggest that UCHL1-KRT18 may contribute to glioma radioresistance in association with stemness maintenance.

## 3. Discussion

Therapeutic resistance remains a major clinical hurdle in glioma management, underscoring the urgent need to elucidate its underlying molecular mechanisms [29,30]. This study identifies the deubiquitinating enzyme UCHL1 as an important contributor to radioresistance in glioma. Clinical data showed that UCHL1 is markedly overexpressed in radioresistant glioma tissues, and its expression correlates closely with poor patient prognosis and tumor recurrence. Similarly, the stemness marker SOX2 showed a comparable expression pattern in radioresistant cells. Mechanistically, UCHL1 bound directly to KRT18 and specifically removed K48–linked polyubiquitin chains, thereby inhibiting proteasomal degradation. This stabilization was accompanied by upregulation of SOX2–mediated stemness properties and an altered cellular response to DNA damage, which together may contribute to radioresistance. Functional rescue experiments indicated that KRT18 knockdown attenuated the UCHL1 overexpression–induced upregulation of SOX2, reduced stemness, and the radioresistant phenotype, suggesting that KRT18 acts as a downstream effector of UCHL1–mediated radioresistance.

Aberrant UCHL1 expression has been documented in multiple malignancies, yet its functional repertoire extends beyond simple substrate stabilization, exhibiting context–dependent oncogenic or tumor–suppressive roles across different tissues [31]. This tissue specificity is well illustrated by the divergent findings across tumor types: in hepatocellular carcinoma, UCHL1 is frequently silenced and acts as a functional tumor suppressor [32], whereas in non–small–cell lung cancer, UCHL1 deubiquitinates and stabilizes Twist1, promoting epithelial–mesenchymal transition [28]; conversely, in triple–negative breast cancer, it sustains cancer stem cell maintenance and self–renewal through the deubiquitination–mediated stabilization of oncoproteins [25]. In pediatric high–grade glioma, UCHL1 has been associated with stem–like cancer cell functions and Wnt/β–catenin activity [33], consistent with the oncogenic direction observed in our glioma models. However, the role of UCHL1 in glioma radioresistance has remained unclear. Our study addresses this gap by demonstrating that UCHL1 is markedly upregulated in radioresistant GBM cell models and that its high expression is associated with adverse clinical outcomes. Notably, our previous study reported that UCHL1 promotes temozolomide resistance in GBM cells by inhibiting the ubiquitination–mediated degradation of keratin 8 [27], suggesting that UCHL1 may modulate responses to both irradiation and chemotherapy. Importantly, beyond its well-characterized substrate specificity, the catalytic activity of UCHL1 has garnered translational interest; small-molecule inhibitors such as LDN–57444 have shown preclinical efficacy in modulating tumor cell behavior [34,35,36]. Nevertheless, the clinical translation of these inhibitors in glioma may be hindered by the challenges of crossing the blood–brain barrier and the potential on–target toxicity to neural progenitor cells [37]. Our findings suggest that combining UCHL1 inhibition with radiotherapy could disable the DDR machinery specifically in GBM stem-like cells, but this strategy warrants cautious optimization to spare normal brain parenchyma. This is particularly relevant given that UCHL1 expression increases with WHO grade and is elevated in glioma patient tissues, supporting its accessibility and clinical relevance as a target.

Identifying KRT18 as a key downstream effector of UCHL1 in glioma radioresistance conceptually advances our understanding of the interplay between intermediate filament proteins and stemness regulation. Previous studies on KRT18 in glioma have predominantly focused on genomic alterations: somatic mutations of *KRT18* are enriched in astrocytomas but rare in glioma, with no subtype-specific expression difference reported [38]. In other cancer types, KRT18 has been functionally characterized as an oncogene; for instance, its overexpression in colorectal cancer correlates with aggressive clinicopathological features and promotes malignant phenotypes including cell viability, migration, and invasion [39,40]. However, the role of KRT18 in regulating cancer stem cell properties remains controversial. In hepatocellular carcinoma and certain epithelial–derived tumors, KRT18 serves as a differentiation marker [41], and its downregulation enhances stemness-like phenotypes and induces EMT [42]. Conversely, in cholangiocarcinoma, KRT18 overexpression promotes proliferation and maintains stemness [43]; similarly, in colorectal cancer, KRT18 is frequently overexpressed in CD44–positive cancer stem cells, and its expression levels correlate with stemness properties and chemotherapy resistance [44]. These findings are consistent with our observations, whereas the opposite direction reported in hepatocellular carcinoma indicates that KRT18–mediated regulation of stemness is markedly tumor–type dependent. However, previous mechanistic studies have largely focused on transcriptional or expression levels. In this study, we show that the pro–stemness function of KRT18 in glioma is dynamically regulated by UCHL1–mediated deubiquitination. UCHL1 removes K48–linked polyubiquitin chains from KRT18, inhibiting proteasomal degradation and sustaining protein stability, which, in turn, contributes to radioresistance. These data suggest a role for UCHL1 in modulating radiotherapy resistance associated with KRT18 stabilization; however, further studies are needed to confirm a direct causal link. This finding highlights the potential role of post–translational modification in KRT18–mediated stemness regulation and provides a possible molecular explanation for the tumor type–dependent functions of KRT18. Building on these findings, our study defines an upstream regulatory event in which UCHL1 promotes SOX2–mediated stemness maintenance through KRT18 stabilization, providing a mechanistic framework for understanding UCHL1’s role in glioma radioresistance.

Despite the established importance of the UCHL1–KRT18 axis in glioma radioresistance, several limitations warrant consideration. First, our conclusions are based mainly on in vitro systems. The colony formation assay, although the gold standard for assessing intrinsic radiosensitivity, does not always predict clinical radioresponse, and our monocellular spheroid model does not capture the cellular heterogeneity or stromal compartment of patient tumors. In particular, cancer–associated fibroblasts can influence radioresistance through paracrine signaling [45], extracellular matrix remodeling, and enhancement of DNA repair, and these contributions were not addressed. In addition, normal human astrocytes were not included as non-malignant controls, and in vivo validation is still lacking. Therefore, our findings should be interpreted as estimates of tumor cell–intrinsic radiosensitivity rather than direct clinical predictors. Second, regarding the mechanistic depth, we cannot exclude contributions from other UCHL1 substrates or parallel resistance pathways, as our study did not systematically interrogate them. Although the catalytically inactive UCHL1 C90S mutant confirmed the requirement for deubiquitinating activity, mutant experiments specifically blocking UCHL1–KRT18 binding were not performed. Moreover, the molecular interface through which KRT18 regulates SOX2 remains to be dissected using chromatin immunoprecipitation, reporter assays, or protein stability analyses, and the reciprocal KRT18 rescue experiment in UCHL1–knockdown cells was not completed. Third, regarding the clinical data, our patient cohort was limited in size, and the survival analyses did not account for concomitant therapies such as extent of resection, temozolomide regimens, or salvage treatments; thus, treatment heterogeneity may introduce unadjusted confounding. Accordingly, the association between UCHL1–KRT18 expression and radiotherapy resistance should be regarded as supportive correlative evidence requiring validation in larger, multi-institutional cohorts with complete treatment records before any clinical translation can be considered.

In summary, this study identifies a mechanism by which UCHL1 promotes glioma radioresistance through deubiquitination–mediated stabilization of KRT18, which in turn upregulates SOX2–mediated stemness features and modulates the cellular response to DNA damage. Given that UCHL1 possesses a druggable catalytic domain, developing specific small-molecule inhibitors of UCHL1 in combination with radiotherapy may represent a potential therapeutic strategy to overcome radioresistance in glioma.

## 4. Materials and Methods

### 4.1. Cell Culture

Human GBM cell lines U251, T98G, LN229, U87MG, along with human embryonic kidney cells 293T, were purchased from the Cell Bank of the Chinese Academy of Sciences (Shanghai, China). All cell lines were authenticated by short tandem repeat (STR) profiling and routinely tested negative for mycoplasma contamination. Cells were cultured in high-glucose Dulbecco’s Modified Eagle Medium (DMEM; #E600002–0500, Sangon Biotech, Shanghai, China) supplemented with 10% fetal bovine serum (FBS; #S711–001S, LONSERA, Montevideo, Uruguay) and 1% penicillin–streptomycin (PS; #15070063, Gibco, Grand Island, NY, USA). All cells were maintained at 37 °C in a humidified incubator containing 5% CO_2_.

### 4.2. Establishing Radioresistant GBM Cell Lines

U251 and LN229 cells in the exponential growth phase were seeded into T25 culture flasks. Upon attachment and reaching 80% confluence, the cells were exposed to 4 Gy of X–ray irradiation. The culture medium was replaced every two days, and cells were passaged when they reached approximately 80% confluence. A second 4 Gy irradiation was administered 24 h after each passage. This irradiation–passage cycle was repeated 15 times over a period of four months, resulting in a cumulative radiation dose of 60 Gy. Following the final irradiation, the cells were passaged an additional 2–3 times to allow phenotypic stabilization, yielding the radioresistant sublines designated U251R and LN229R. Throughout the entire procedure, the parental (non-irradiated) U251 and LN229 cells were cultured and passaged in parallel under identical conditions, except for the omission of irradiation, and served as control cultures.

### 4.3. Glioma Specimens

Glioma tissue specimens were collected from patients at the Children’s Hospital of Soochow University (Suzhou, China). Relevant clinical parameters, including age, sex, tumor subtype, and treatment history, were recorded for subsequent statistical analyses. Radiosensitivity and radioresistance were classified by serial post-radiotherapy imaging. The first pre-radiotherapy magnetic resonance imaging (MRI) served as the baseline. Progression was defined per the response assessment in neuro-oncology (RANO) 2.0 as a ≥25% increase in the sum of products of perpendicular diameters, or a ≥40% increase in volume, relative to baseline, with pseudoprogression excluded [46]. Radioresistant cases had confirmed progression; radiosensitive cases showed stable or decreased tumor burden.

### 4.4. Database Analysis

The GEO2R database (https://www.ncbi.nlm.nih.gov/geo/geo2r/ (accessed on 1 June 2025)) was employed to retrieve gene expression datasets and identify differentially expressed genes. The GEO datasets GSE199862, GSE207002, and GSE140746 were selected to investigate genes associated with radiotherapy resistance in GBM cells; detailed information on the cell models used to generate these data is provided in Supplementary Table S1. The GEPIA2 database (http://gepia2.cancer-pku.cn/) and Chinese Glioma Genome Atlas (CGGA) database (https://www.cgga.org.cn/) were utilized to evaluate the correlation between target gene expression levels and patient prognosis. UCHL1 expression data across glioma cell lines were obtained from the CCLE database [47].

### 4.5. Transient Transfection and Lentiviral Infection

For transient transfection, cells were seeded in 6–well plates and transfected with plasmid DNA using Lipofectamine^®^ 3000 (#L3000075, Invitrogen, Carlsbad, CA, USA) at a DNA–to–reagent ratio of 1:1 (μg:μL) according to the manufacturer’s instructions. For stable gene manipulation, lentiviral particles were introduced into cells cultured in 6–well plates. Polybrene was added to enhance transduction efficiency. After 24 h of incubation, the medium was replaced, and stably transduced cells were selected using puromycin (2.5 μg/mL; #ST551, Beyotime, Shanghai, China). The shRNA sequences targeting human *UCHL1* and *KRT18* are listed in Table 1.

### 4.6. Quantitative Real-Time PCR (qRT-PCR)

Total RNA was extracted from cultured cells using the RNA extraction kit (#RC102, Vazyme, Nanjing, China) following the manufacturer’s instructions. Complementary DNA (cDNA) was then synthesized from equal amounts of purified RNA using the PrimeScript RT Kit (#R323–01, Vazyme). PCR was performed with qPCR Master Mix Kit (#Q712–02, Vazyme) on a suitable real-time PCR system. Melting curve analysis was performed after each amplification run, and the melting curves are shown in Supplementary Figure S5. Relative mRNA expression was normalized to that of *GAPDH*. The specific primer sequences for target genes are provided in Table 2.

### 4.7. Immunohistochemistry (IHC)

Glioma tissue specimens were fixed in 10% neutral-buffered formalin, embedded in paraffin, and sectioned at 4 μm thickness using a microtome. Sections were deparaffinized in xylene and rehydrated through a graded ethanol series to distilled water. Antigen retrieval was performed by heating the sections in citrate buffer (pH 6.0) using a microwave or pressure cooker, following standard protocols. Endogenous peroxidase activity was blocked by incubation with 3% H_2_O_2_ for 10 min at room temperature. After blocking nonspecific binding sites, sections were incubated with primary antibodies overnight at 4 °C. The primary antibodies used in this study were as follows: UCHL1 antibody (1:200, #66230–1–Ig, Proteintech, Wuhan, China); KRT18 antibody (1:250, #4548, Cell Signaling Technology, Danvers, MA, USA). Antigen–antibody complexes were detected using horseradish peroxidase (HRP)–conjugated secondary antibodies, with 3, 3′–diaminobenzidine (DAB) as the chromogenic substrate. Sections were counterstained with hematoxylin, dehydrated, mounted, and examined under a microscope.

### 4.8. Immunofluorescence (IF)

Cells were fixed with 4% paraformaldehyde and blocked with 1% FBS for 30 min at room temperature. Subsequently, cells were incubated overnight at 4 °C with primary antibody against SOX2 (1:200, #23064, Cell Signaling Technology, Danvers, MA, USA). After washing, cells were incubated with species-specific fluorophore–conjugated secondary antibody Alexa Fluor 488 goat anti-rabbit IgG (H + L) (1:500, #A32731, Thermo Fisher Scientific, Waltham, MA, USA) for 45 min at room temperature. Nuclei were counterstained with DAPI (1:1000, #D9542, Sigma Aldrich, St. Louis, MO, USA). Fluorescence images were captured using a fluorescence microscope (Carl Zeiss, Jena, Germany).

### 4.9. Western Blot

Cells were lysed in RIPA buffer (#P0013C, Beyotime) supplemented with a protease and phosphatase inhibitor cocktail. Protein concentrations were determined using a BCA protein assay kit (#P0011, Beyotime). Equal amounts of protein were separated by sodium dodecyl sulfate–polyacrylamide gel electrophoresis (SDS–PAGE) and transferred onto polyvinylidene difluoride (PVDF) membranes (#88518, Merck Millipore, Billerica, MA, USA). Membranes were blocked with bovine serum albumin (BSA) for 1 h and incubated with primary antibodies overnight at 4 °C. Following washes with Tris-buffered saline containing Tween 20 (TBST), the membranes were incubated with HRP–conjugated secondary antibodies (Anti–rabbit, 1:2000, #7074; Anti–mouse, 1:2000, #7076, Cell Signaling Technology) for 1 h at room temperature. Protein bands were visualized using an enhanced chemiluminescence (ECL) detection system, with GAPDH serving as the loading control. The primary antibodies used were as follows: anti–UCHL1 (1:1000, #13179, Cell Signaling Technology), anti–KRT18 (1:1000, #AG8331, Beyotime), and anti–SOX2 (1:1000, #23064, Cell Signaling Technology).

### 4.10. Co-Immunoprecipitation (Co-IP)

Cells were lysed in NP-40 buffer supplemented with 1% protease and phosphatase inhibitor cocktail. To reduce nonspecific binding, lysates were pre–cleared by incubation with magnetic beads (#22202, Beaver, Suzhou, China). Subsequently, 5 μL of primary antibody or control IgG was added to 200 μg of lysate (1 μg/μL), and the mixture was incubated overnight at 4 °C with gentle rotation. Magnetic beads were then added, and the incubation continued for an additional 3 h at 4 °C. The immunoprecipitated complexes were washed, eluted, and subsequently analyzed by immunoblotting.

### 4.11. Mass Spectrometry (MS)

To identify potential UCHL1–interacting proteins, immunoprecipitated samples from Flag–UCHL1–expressing cells were eluted, digested into peptides, and subjected to liquid chromatography–tandem mass spectrometry (LC–MS/MS). Briefly, the immunoprecipitated products were separated by SDS–PAGE and visualized by Coomassie Brilliant Blue staining. Peptide mixtures were extracted from the gel bands, dried, and analyzed using LC–MS/MS. Database searches were performed against the UniProtKB/Swiss–Prot database (*Homo sapiens*; reviewed entries only) available at https://www.uniprot.org/.

### 4.12. Irradiation Condition

Cells were irradiated using a 160 kV X–ray linear accelerator (RS–2000 Pro, Siemens, Malvern, PA, USA) at a dose rate of 198 cGy/min. After irradiation, cells were returned to the incubator, and samples were collected at the indicated time points.

### 4.13. Clonogenic Survival Assay

Adherent cells in the logarithmic growth phase were trypsinized with 0.25% trypsin–EDTA and centrifuged at 1200 rpm (Eppendorf Centrifuge 5810 R, rotor A–4–62, radius 17.1 cm) for 5 min to obtain single–cell suspensions. Cell viability and density were determined using a hemocytometer. Cells were seeded at gradient densities into 6–well plates and allowed to attach for 24 h, followed by irradiation with graded doses (0–8 Gy). After irradiation, cells were cultured for 10 days. Once visible colonies appeared, the culture was terminated. Cells were washed three times with PBS, fixed with 4% paraformaldehyde for 15 min at room temperature, and stained with 0.1% crystal violet for 15 min. Plates were then air–dried for 24 h at room temperature.

### 4.14. Sphere Formation Assay

For serum–free stem cell culture, cells were cultured in DMEM/F12 medium supplemented with 1× penicillin/streptomycin, 10 ng/mL recombinant human basic fibroblast growth factor (bFGF), 10 ng/mL epidermal growth factor (EGF), and B27 supplement. For the sphere formation assay, adherent cells at 80% confluence were washed twice with pre–warmed PBS and trypsinized. Digestion was neutralized with serum–containing medium, and cells were centrifuged at 1200 rpm (Eppendorf Centrifuge 5810 R, rotor A–4–62, radius 17.1 cm) for 5 min. Cells were resuspended in serum–free medium and adjusted to a density of 1500 cells/mL. Aliquots of 200 μL of the cell suspension (300 cells/well) were seeded into ultra-low attachment 96–well plates, with 10 technical replicates per condition. Plates were incubated under static conditions at 37 °C in 5% CO_2_ and 95% humidity. To avoid disturbing sphere aggregation, plates were not moved for the first 48 h. Medium was replaced every 72 h by carefully removing half of the volume (100 μL) and replenishing with pre–warmed fresh medium. Once spheres reached a diameter of ≥50 μm, medium changes were performed using a 27 G needle to minimize sphere loss. After 7 days of culture, sphere-forming capacity was quantified by computer-assisted image analysis, with spheres larger than 100 μm in diameter scored as positive [48,49].

### 4.15. Extreme Limiting Dilution Analysis (ELDA)

To assess tumor self–renewal capacity, cells were resuspended as homogeneous single–cell suspensions, and viable cell counts were determined using a hemocytometer. Cells were seeded at various densities (100, 50, 20, 10, 5, and 1 cell per well) into ultra–low attachment 96–well plates, with 10 replicate wells per density. Plates were incubated at 37 °C in a humidified incubator containing 5% CO_2_. On day 7, wells containing one or more spheres with a diameter greater than 50 μm were scored as positive. Data were analyzed using the online ELDA tool (http://bioinf.wehi.edu.au/software/elda/ (accessed on 1 June 2025)) to determine stem cell frequency and generate corresponding statistical plots.

### 4.16. Comet Assay

For the alkaline comet assay, adherent cells were gently rinsed with ice–cold PBS, harvested, and centrifuged at 800 rpm (Eppendorf Centrifuge 5810 R, rotor A–4–62, radius 17.1 cm) for 5 min. After three washes with cold PBS, cells were resuspended at a density of 1 × 10^7^ cells/mL and kept on ice. A three–layer agarose gel system was prepared on pre–warmed slides. A base layer of 1% normal–melting–point agarose (NMA) in PBS was applied to frosted slides and allowed to solidify at 4 °C. Subsequently, 10 μL of cell suspension was gently mixed with 75 μL of 0.7% low–melting–point agarose (LMA), layered onto the base gel, covered with a coverslip, and allowed to solidify at 4 °C. A third protective LMA layer (75 μL) was applied to seal the sample. After solidification, the slides were immersed in ice–cold lysis buffer containing 10% DMSO and incubated for 1.5 h at 4 °C in the dark. Slides were then placed in freshly prepared alkaline electrophoresis buffer (300 mM NaOH, 1 mM EDTA) and allowed to unwind for 40 min at 4 °C. Electrophoresis was performed at 25 V and 300 mA for 25 min at 4 °C under dim light. Thereafter, slides were neutralized three times in 0.4 M Tris–HCl (pH 7.5) and stained with 20 μg/mL propidium iodide for 15 min at room temperature in the dark. Comet images were acquired using a fluorescence microscope. For quantification, all cells within one randomly selected field of view per group were counted under the fluorescence microscope, and the percentage of comet-positive cells was calculated from this cell population.

### 4.17. Statistical Analysis

Data are presented as the mean ± standard error of the mean (SEM) from at least three independent experiments. Quantification was performed in a double–blind manner. Statistical analyses were performed using GraphPad Prism 10.0. Comparisons between two groups were analyzed using an unpaired Student’s *t*–test, whereas comparisons among multiple groups were assessed using one-way analysis of variance (ANOVA). Survival curves were generated using the Kaplan–Meier method, and statistical significance was evaluated using the log–rank test. A *p*–value of < 0.05 was considered statistically significant. In the figures, significance is denoted as * *p* < 0.05, ** *p* < 0.01, or *** *p* < 0.001.

## Figures and Tables

**Table 1:** Sequences for UCHL1 shRNA and KRT18 shRNA.

Name	Sequence
shNC	5′-CCTAAGGTTAAGTCGCCCTCG−3′
*UCHL1*-shRNA	5′-CGGGTAGATGACAAGGTGAAT−3′
*KRT18*-shRNA	5′-CTTCATGAAGAAGAACCACGA−3′

**Table 2:** Primers for target genes and GAPDH.

Gene	Sequence	
*UCHL1*	forward	5′-CCTGTGGCACAATCGGACTTA-3′
	reverse	5′-CATCTACCCGACATTGGCCTT-3′
*GAPDH*	forward	5′-GGAAGATGGTGATGGGATT-3′
	reverse	5′-GGATTTGGTCGTATTGGG-3′
*KRT18*	forward	5′-CCACACAGTCTGCTGAGGTT-3′
	reverse	5′-TCCAAGCTGGCCTTCAGATT-3′
*SOX2*	forward	5′-AAGGATAAGTACACGCTGCCC-3′
	reverse	5′-GTTCATGTGCGCGTAACTGT-3′

## Data Availability

The original contributions presented in this study are included in the article/Supplementary Material. Further inquiries can be directed to the corresponding author.

## Supplementary Materials

The following supporting information can be downloaded at: https://www.mdpi.com/article/10.3390/ijms27219875/s1.
