# Supplementary material for: The Deubiquitinating Enzyme UCHL1 Promotes Radiotherapy Resistance in Glioma by Stabilizing Keratin 18
Source: Int J Mol Sci. 2026 Nov 4;27(21):9875. doi: 10.3390/ijms27219875 (PMC13764221; doi:10.3390/ijms27219875)
Supplement: Supplementary file 1 [file ijms-27-09875-s001.zip › ijms-4624288-supplementary.pdf]

# Supplementary Figures

## Figure S1

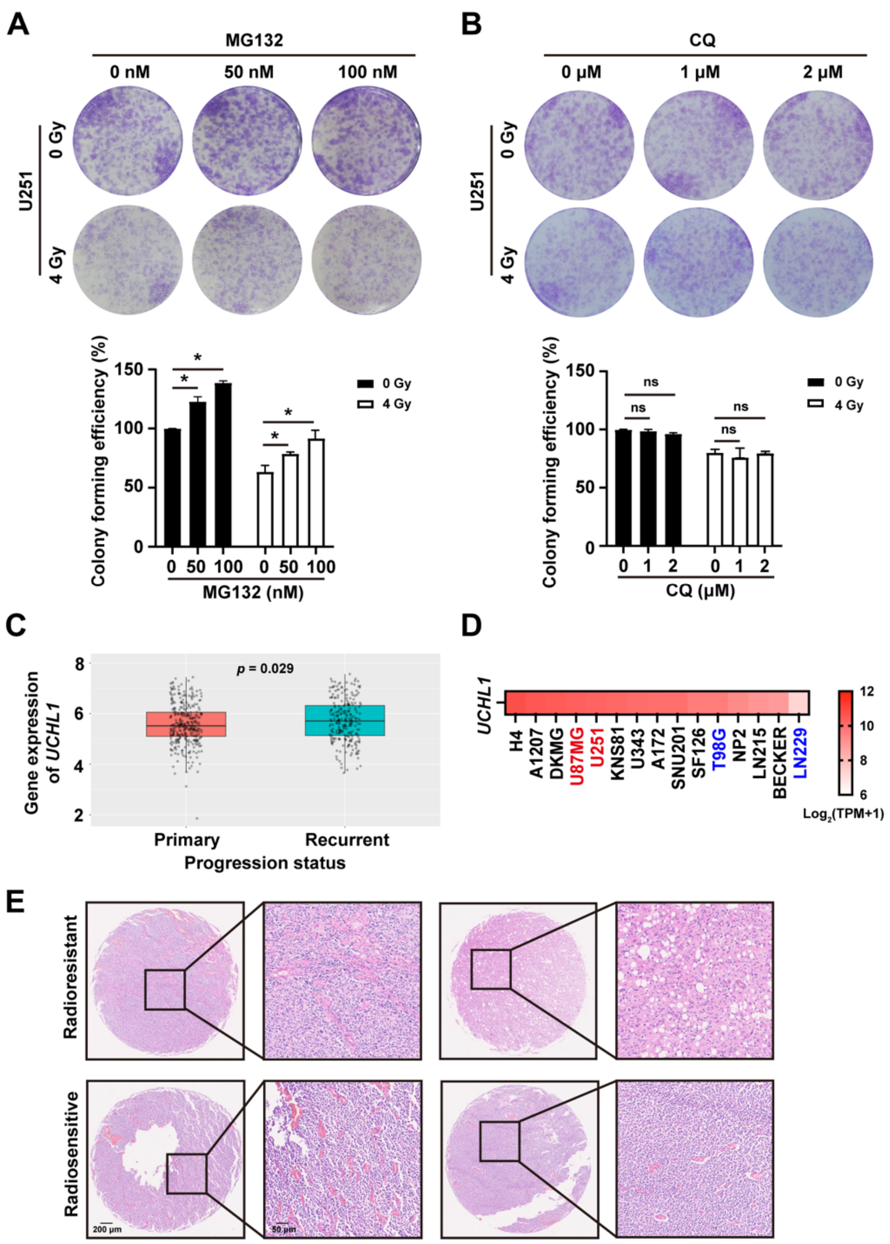


**Figure S1. The ubiquitin–proteasome system mediates GBM radioresistance and identifies UCHL1 as a key component. (A–B)** Clonogenic survival assays evaluating the colony-forming capacity of U251 cells treated with increasing concentrations of MG132 (0, 50, and 100 nM) or CQ (0, 1, and 2 μM) in combination with 4 Gy irradiation (n = 3). **(C)** *UCHL1* expression in primary versus recurrent glioma specimens from the CGGA dataset. **(D)** *UCHL1* expression levels across

glioma cell lines in the CCLE database. **(E)** Hematoxylin and eosin (H&E) staining of radiosensitive and radioresistant tumor tissues in patients with glioma. * $p < 0.05$, ns, not significant.

## Figure S2

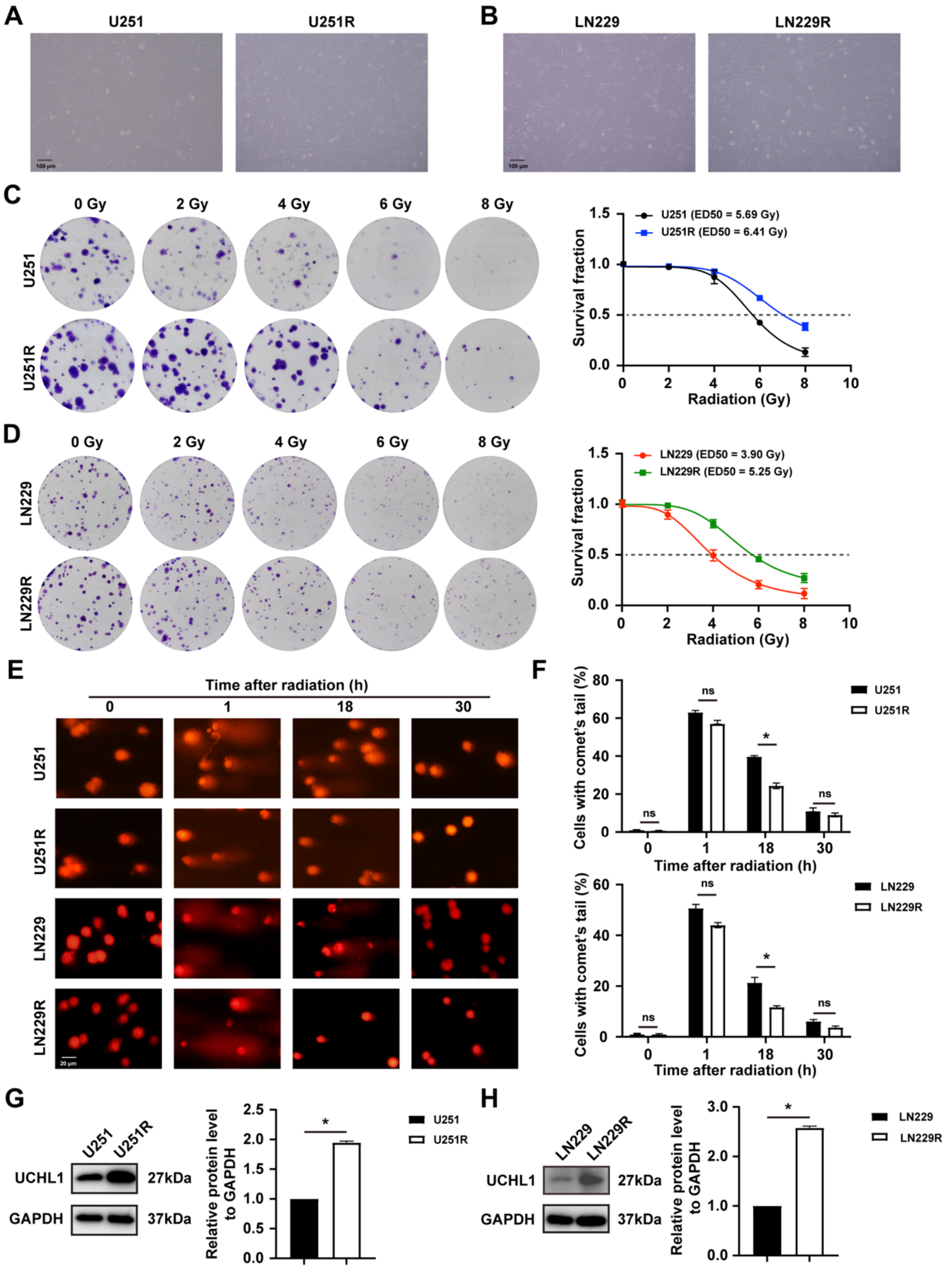


**Figure S2. Radioresistant GBM sublines exhibit enhanced DNA repair capacity and**

upregulated UCHL1 expression. (A–B)** Representative light microscopy images of U251, U251R, LN229, and LN229R cells. Scale bar: 100 μm. **(C–D)** Clonogenic survival assays evaluating the colony-forming capacity of the indicated cell lines, with quantification of colony numbers and dose survival curves (n = 3). **(E–F)** Comet assay analysis of DNA damage repair capacity in the indicated cell lines following irradiation (n = 3). **(G–H)** Western blot analysis of UCHL1 protein expression levels in the indicated cell lines (n = 3). * $p < 0.05$, ns, not significant.

# Figure S3

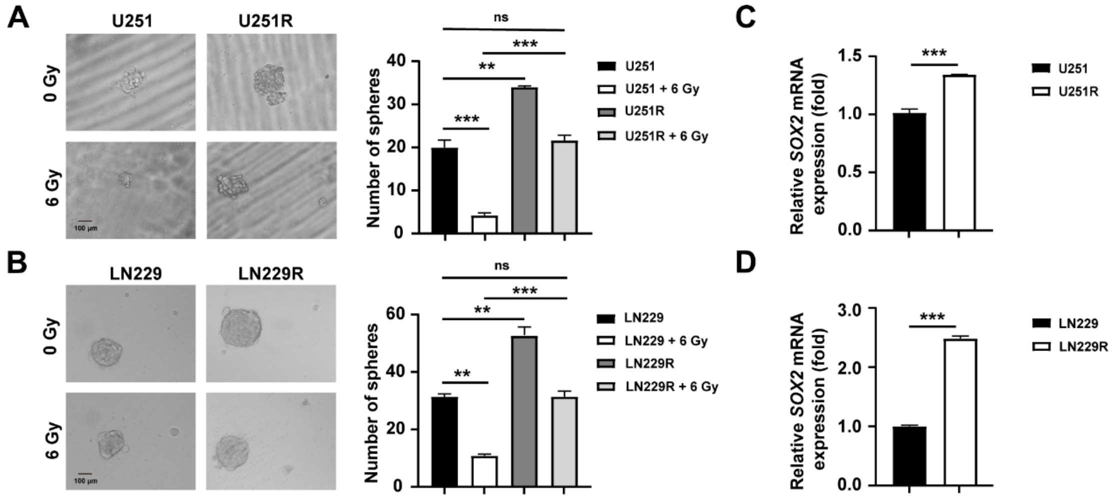


**Figure S3. Radioresistant GBM sublines exhibit enhanced stemness properties. (A–B)** Sphere formation assays assessing the size and number of tumor spheres formed by U251, U251R, LN229, and LN229R cells with or without 6 Gy irradiation. Scale bar: 100 μm (n = 10). **(C–D)** qRT-PCR analysis of SOX2 mRNA levels in the indicated cell lines (n = 3). ** $p < 0.01$, *** $p < 0.001$, ns, not significant.

## Figure S4

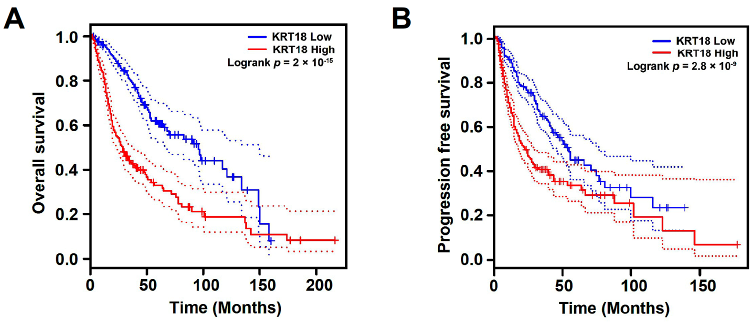


**Figure S4. Association between *KRT18* Expression and survival outcomes in glioma patients.** Kaplan–Meier survival analysis of the correlation between KRT18 expression and overall survival **(A)** and progression-free survival **(B)** in glioma patients. Survival curves were compared using the log-rank test.

## Figure S5

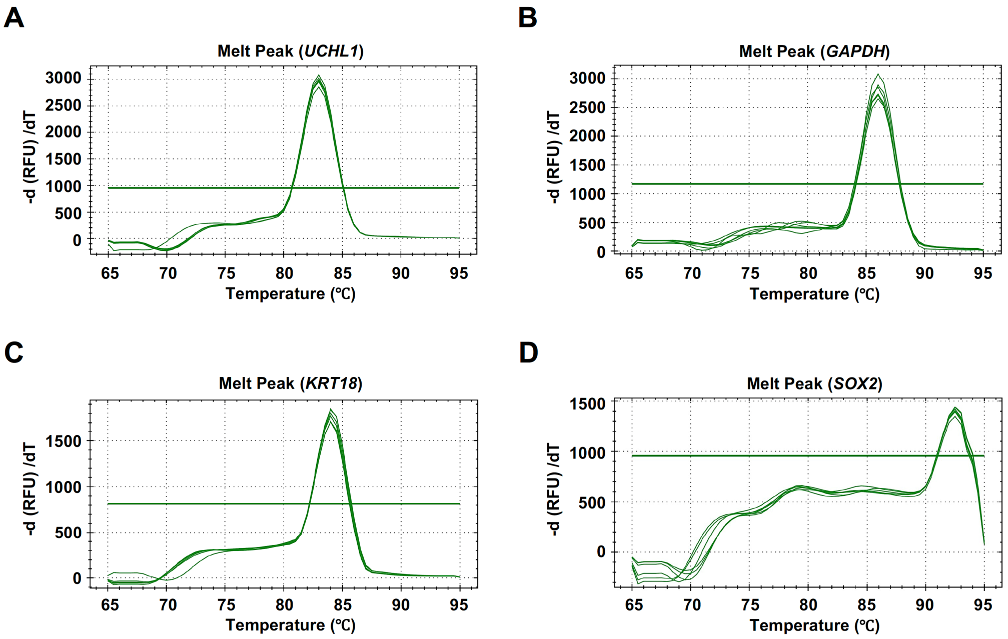


**Figure S5. Specificity verification of qRT-PCR primers by melting curve analysis.** Melting curve analysis of qRT-PCR products for **(A)** *UCHL1*, **(B)** *GAPDH*, **(C)** *KRT18*, and **(D)** *SOX2*. Each panel shows a single sharp peak, indicating specific amplification of the target gene without nonspecific products or primer–dimer formation.

**Supplementary Table**

Table S1 Experimental models in the GEO datasets used for analysis

| GEO accession | GSE199862 | GSE207002 | GSE140746 |
|---|---|---|---|
| Parental cell line | U373 | U87MG | 1095 |
| IR Survivor cell line | U373-60Gy | U87MG-RR | 1095_IR |
| Total dose | 60 Gy | 30 Gy | 14 Gy |
| Treatment protocol | Cells were exposed to 2 Gy ionizing radiation every day for 6 weeks until the total dose reached 60 Gy | Cells were subcutaneously or orthotopically implanted in BALB/c nude mice, repeatedly irradiated (2 Gy per fraction, 30 Gy total), and the surviving radioresistant cells were then isolated | Cells were exposed to 2 Gy X-ray until the total dose reached 14 Gy |
